# Meeting Reports: The Pittsburgh Conference

**DOI:** 10.1155/S146392468100028X

**Published:** 1981

**Authors:** P. B. Stockwell


### Meeting Reports

### The Pittsburgh Conference

The conference programme this year encompassed over 900 scientific and technical papers. As has become the custom, many are pseudotechnical presentations by instrument company staff describing their latest developments. With the developments in microprocessor applications, many of the papers can be broadly classified as automation, and as such, a complete summary of these is not possible. This report describes a cross section of papers in this category.

Janule [1] described the development of a prototype instrument for measuring surface tension of emulsions and its subsequent commercialisation. The technology used is a refinement of the maximum bubble pressure method. Results are stable and repeatable to within ± ½%, with the direct digital control being provided through the computer interface.

De Souza [2] described how the use of continuous or automated analytical instruments cuts back on manpower requirements and gives real-time analysis. However, rigid maintenance becomes necessary because of the complexity in operating some of these sophisticated pieces of equipment. Analytical devices for measuring sulphur gas concentrations in gas streams are available on the market, the most notable of these are based on the principles of flame photometry, ultra-violet spectroscopy and electrochemical sensing. The advantages of each group were discussed. Detectors for measuring carbon dioxide, oxygen, nitrogen oxides plus particulates were also discussed with reference to their application to the process industry.

A continuous oxygen analysis system which has operated satisfactorily on coke oven gas sampled immediately after the booster pumps in the coke plant was described by Manka [3]. The gas glows at sufficient pressure without the use of pumps through a moisture trap, a naphthalene removal system, and a filter to an oxygen analyser which depicts the oxygen content as a peak on the recorder each time an oven is charged with coal. Two methods for charging coal into the ovens were discussed which maintains the oxygen level in coke oven gas at or below 1.0%. Malmstadt [4] used his considerable experience of instrumentation to discuss how recent developments in automated instrumentation influences the general types of analytical systems used for process control. In one system the process control laboratory is located near the process, and the lab's temperature and humidity are controlled. The samples are transported to the lab, and then automated instruments are used that are similar to the instruments found in a conventional analytical laboratory. It was shown how low-cost microcomputers greatly improve the potential for a compact and completely automated process control laboratory. Spectrometers, colorimeters, stopped-flow analysers for kinetic and equilibrium chemical methods, polarographs, sample prep units, and many other conventional laboratory instruments that are redesigned for greater reliability and complete automation are all candidates for automated process analyses.

Stockwell [5] reviewed current developments in automation of laboratory methods, with emphasis on the managerial outlook demanded from the modern analytical chemist and on the underlying philosophy of automation. Problems of education, communication and specification highlighted by these inter-related problems were discussed in detail. Some recent instrumental developments and the role of microprocessors, their advantages and limitations were discussed. Details of a short course devoted to automated chemistry were presented and contrasted to those courses available in the USA.

Whilst sophisticated analytical instruments dominate laboratory automation, some of the workhorse analysers in the process industry are all non-specific, rugged devices noted for their reliability. Oliver [6] described vapour pressure measurement, density/specific gravity, conductivity, viscosity and refractive index, and their applications.

Jirauch [7] described a system to increase the throughput of a multiple FIA system. A microprocessor data reduction and control system is used, and the system allows four different analyses to be made simultaneously for each sample. The system can be extended to handle multi-samplers with multi-channels attached to each sampler. Each sample can be controlled, started, and stopped independently of any other sampler. Hence, each sampler can be an entity in itself and act as though it were the only sampler being controlled by the system. Ranger [8] presented an overview of simplex optimisation as applied to FIA. Details of the computer programme to perform these analyses are available from the author.

Schick [9] outlined the advantages of FIAtron's microprocessor controlled solution handling system. The SHS-200 utilises the many advantages of flow injection methodology. The heart of the SHS-200 is a dual channel sample injector, an all teflon device consisting of electrically driven four-way solenoid valves of micro-miniature construction. A programmable reagent valve and an optically encoded four-channel persistaltic pump as well as an optional sample changer are also part of the SHS-200. All components are under software control and are readily programmable from the front panel keyboard. Laminar flow theory provides simple expressions for the appearance times and peak widths of samples in simple FIA systems as a function of the parameters of the system and the diffusion coefficient of the sample. These expressions have been used by Vanderslice [10] to illustrate the effect of varying the parameters. A simple FIA system was assembled and solvents of different viscosity were used to vary the diffusion coefficient of the system and resulting changes in the dispersion were measured.

Stewart [11] described how the use of two phase systems in FIA and in automated multiple flow injection analysis (AMFIA) greatly expands the usefulness of these unsegmented continuous flow systems. The two phases are separated by surface tension and gravity, and in a special separator. It was not necessary to use peristaltic pumps to remove the two streams from the separator. The concepts of the system were tested by the extraction of sulphosalicylic acid from an aqueous system into a N-butanol system.

Stewart [12] also discussed the use of an exponential dilution chamber as a means of scale expansion in FIA. The concept has been tested with colorimetric sugar analysis, colorimetric acid base analysis, colorimetric phosphate determinations, flame emission sodium determinations, fluorescent leucine determinations, conductance salt determinations, and potentiometric hydrogen ion determinations.

Weinberger [13] described various preparative techniques available to the user, so-called "unit operations". These include dissolution, dilution, dialysis, derivitisation, distillation, evaporation, extraction and filtration. Modules are commerically available to accomplish any or all of these tasks. Benefits obtained from this family of techniques, such as faster rate of analysis, unattended operation, lowered reagent and glassware conumption increased precision were discussed.

Baulu [14] described an automated dual channel extraction system which is designed to avoid difficulties inherent in handling particulate-containing samples with conventional pumping and valving modules. Extraction is accomplished by segmented sample/solvent flow through Teflon tubing wound in a double helix. Fluid transfer power is supplied by compressed air, whilst the valving system is exposed solely to "clean" fluids – air and methylene chloride. Use of an appropriate phase separator permits other water/solvent combinations to be used.

Massie [15] has developed a multi-channel, continuous flow instrument for use in water testing laboratories. This instrument is capable of running six tests simultaneously, many of the tests in the part-per-billion range. The automatic sampler has a special feature so that it can accommodate samples with different matrices, such as acid digested samples for a TKN determination. The programmer is capable of interfacing with two samplers; therefore, four different sample matrices can be handled at one time.

Conetta [16] presented a technique whereby total phosphate in water samples may be determined by a photochemical decomposition of organic phosphorus compounds and the thermal hydrolysis of acid-hydrolysable phosphates followed by the conventional colorimetric determination of the liberated ortho-phosphate with molybdenum blue. Analytical performance data were presented and discussed.

Further details of these papers can be obtained by corresponding with the primary authors. Many of these have indicated that they will be submitting their manuscripts to this Journal for formal publication.

*P.B. Stockwell*

REFERENCES
[1] A continuous in-process surface tension measuring device. *V.P. Janule,* Madison-Kipp Corporation, 201 Waubesa Street, Madison, Wisconsin 53704.
[2] Automation in monitoring gases and particulates in the pulp and paper industry. *T.L.C. De Souza,* Pump & Paper Research Institute of Canada (PPRIC), 570 St. John's Blvd., Pointe Claire, Que., Canada.
[3] Continuous analysis of oxygen in coke oven gas. *Dan P. Manka,* Consultant, 1109 Lancaster Avenue, Pittsburgh, Pennsylvania 15218.
[4] Automated instrumentation for chemical analyses in process control. *H.V. Malmstadt,* School of Chemical Sciences, Univ. of Illinois, Urbana, Illinois 96740.
[5] The changing face of laboratory automation: Present & Future. *P.B. Stockwell,* Editor, Journal of Automatic Chemistry, United Trade Press, 33-35 Bowling Green Lane, London, EC1R 0DA.
[6] Non-specific methods of analysis: the unsung heroes of the process control world. *R.T. Oliver,* Foxboro Analytical, Balsbough Electro-Chemical Ctr., Armstrong Road, Plymouth Industrial Park, Plymouth, MA 02360.
[7] A microprocessor-based control and data reduction system for Multi-channel flow injection analysers. *Dorothea H. Jirauch,* Lachat Chemicals Inc., 10500 N. Port Washington Road, Mequeon. WI 53092.
[8] Simplex optimisation applied to flow injection analysis. *Craig R. Ranger,* Lachat Chemicals Inc., Instrument Division, 10500 N. Port Washington Road, Mequon. WI 53092.
[9] Applications of a new, automated solution handling system utilising flow injection methodology. *K.G. Schick,* Fiatron Systems Inc., 6651 N. Sidney Pl., Milwaukee WI 53209.
[10] Guidelines for designing flow injection analysis systems. *Joseph T. Vanderslice,* USDA, Nutrient Composition Lab., Room 225, Building 308, Barc-East, Beltsville, MD 20705.
[11] A model for two phase AMFIA systems. *Kent T. Stewart,* USDA, Nutrient Composition Lab., Room 225, Building 308, Barc-East, Beltsville, MD 20705.
[12] Use of an exponential dilution chamber as a means of scale expansion in flow injection analysis (FIA). *Kent K. Stewart,* USDA, Nutrient Composition Lab., Room 225, Building 308, Barc-East, Beltsville, MD 20705.
[13] Some aspects of automated sample preparation. *Robert Weinberger,* Technicon Industrial Systems, Tarrytown, New York 10591.
[14] A novel, automated liquid extraction technique for aqueous pesticides samples. *P. Baulu,* Pesticides Section, Laboratory Services Branch, Ministry of the Environment, PO Box 213, Rexdale, Ontario, Canada.
[15] A new continuous-flow, highly sensitive, six channel water analyser. *Lynne Massie,* ALPKEM Corporation, PO Box 1260, Clackamas OR 97015.
[16] An automated method for the measurement of total phosphate by UV digestion. *A. Conetta,* Technicon Industrial Systems, Tarrytown, New York 10591.

### Automation in food analysis

A joint meeting on the above topic, organised by the Automatic Methods Group and South East Region of the Analytical Division of the Royal Society of Chemistry, UK, was held in Leatherhead, Surrey on 12 December 1980.

In the morning session Dr Folkes [1] discussed the applications of HPLC in the analysis of foodstuffs; he particularly emphasised the contribution of automation both during the development of analytical procedures and subsequently during their routine use. The second paper was presented by Mr Coverly [2] who discussed the automation of on-line sample preparation procedures for HPLC, with particular reference to solid-liquid extraction, liquid-liquid extraction, concentration, solvent exchange and derivatisation techniques. The potential for the incorporation of these procedures into fully automated analytical systems for use in the food industry was considered. The final paper of the morning session was given by Dr Saxby [3] who briefly described a compact computer-controlled quadrupole mass spectrometer coupled to a gas chromatograph. The application of the technique was illustrated by reference to work on the detection of taints and off-flavours in foodstuffs. The use of the peak-finder mode of operation, in which mass spectra are recorded on all peaks from the GC, was exemplified by reference to the detection of chlorobenzene in milk products. The use of ion-monitor mode, in which only selected ions are monitored, was illustrated by the detection of chloroanisoles, chlorophenols and mesityl oxide in a variety of materials.

After lunch the Automatic Methods Group held a short AGM before the first paper in the afternoon session in which Dr Osborne [4] discussed the application of near IR(NIR) in the automated analysis of protein and moisture levels in cereals and cereal products. He also indicated further possible applications including the measurement of flour colour, degree of starch damage and the prediction of bread-making quality of the flour. The second paper, by Mr Davies [5] considered the use of automated ion-selective electrode analytical procedures with particular reference to the determination of trace chloride levels in poultry meat. Finally, Mr Steele [6] described some automated on-line monitoring systems for the measurement of a variety of physical parameters of food materials during processing. He particularly emphasised the impact of micro-electronics in the fields of weighing, sorting, flow measurement and sizing, and the contribution of microprocessors in the field of integrated plant control systems. Finally he gave a brief insight into the future with a mention of the use of a microprocessor controlled automated NMR spectrometer for the measurement of fat in a chocolate ingredient.

The meeting was well received by the audience and a particularly useful exchange of information and ideas was obtained during the two discussion periods at the end of the morning and afternoon sessions. It was unfortunate that an attendance of 35 did not do justice to the excellent contributions from the speakers and the superb facilities provided by our hosts BFMIRA.

*Clive J. Jackson*

*See page 103 for references*

